# Exploring Cause of Death in Social Convoy Membership: The Case of Pauline

**DOI:** 10.1093/geroni/igaa057.2228

**Published:** 2020-12-16

**Authors:** Sara Stemen, Kate de Medeiros, M Elise Radina

**Affiliations:** Miami University, Oxford, Ohio, United States

## Abstract

People receive support from a fluid convoy of individuals. Historically, convoy membership has been limited to meaningful, living persons. However, research incorporating the continuing bonds model suggests that individuals who have died can also be convoy members as relationships can be preserved through pictures, memories, and after death communication experiences. Building on this idea, this presentation uses a qualitative case study to explore whether (and if so, how) continuing bond relationships are influenced by the way that individuals die. Pauline, a 67 year-old widow, compares the “natural” deaths of her sister and father-in-law to the suicide of her husband. Careful readings of her interview transcript reveal that the unexpected way that her husband died became a salient part of her identity and the way she connects with others. Consequently, this case study provides insights for researchers who may consider cause of death as a potential contributing factor to convoy membership.

